# Adults Meeting Fruit and Vegetable Intake Recommendations — United States, 2013

**Published:** 2015-07-10

**Authors:** Latetia V. Moore, Frances E. Thompson

**Affiliations:** 1 Division of Nutrition, Physical Activity, and Obesity Prevention and Control, National Center for Chronic Disease Prevention and Health Promotion, CDC; 2 Division of Cancer Control and Population Sciences, National Cancer Institute, National Institutes of Health

Eating more fruits and vegetables adds nutrients to diets, reduces the risk for heart disease, stroke, and some cancers, and helps manage body weight when consumed in place of more energy-dense foods (1). Adults who engage in <30 minutes of moderate physical activity daily should consume 1.5–2.0 cup equivalents of fruit and 2–3 cups of vegetables daily.* However, during 2007–2010, half of the total U.S. population consumed <1 cup of fruit and <1.5 cups of vegetables daily; 76% did not meet fruit intake recommendations, and 87% did not meet vegetable intake recommendations (2). Although national estimates indicate low fruit and vegetable consumption, substantial variation by state has been observed (3). Fruit and vegetable intake information from the Behavioral Risk Factor Surveillance System (BRFSS) is the sole source of dietary surveillance information for most states, but frequency of intake captured by BRFSS is not directly comparable to federal intake recommendations, which are expressed in cup equivalents. CDC analyzed median daily frequency of fruit and vegetable intake based on 2013 BRFSS data for the 50 states and the District of Columbia (DC) and applied newly developed prediction equations to BRFSS to calculate the percentage of each state’s population meeting fruit and vegetable intake recommendations. Overall, 13.1% of respondents met fruit intake recommendations, ranging from 7.5% in Tennessee to 17.7% in California, and 8.9% met vegetable recommendations, ranging from 5.5% in Mississippi to 13.0% in California. Substantial new efforts are needed to build consumer demand for fruits and vegetables through competitive pricing, placement, and promotion in child care, schools, grocery stores, communities, and worksites.

BRFSS is an ongoing state-based random-digit–dialed telephone survey of noninstitutionalized, civilian adults aged ≥18 years residing in the United States. BRFSS collects data on health risk behaviors and conditions, chronic diseases and conditions, access to health care, and use of preventive health services and practices related to the leading causes of death and disabilities in the United States (4). BRFSS asks respondents how many times per day, week, or month they consumed 100% fruit juice, whole fruit, dried beans, dark green vegetables, orange vegetables, and other vegetables over the previous month as part of the rotating core questionnaire administered every other year. For these analyses, respondents were excluded if they did not reside in the 50 states or DC, were missing responses to one or more questions, or had implausible reports of fruit or vegetable intake (reported eating fruit >16 times per day or vegetables >23 times per day) (5); after excluding these 118,193 (24%) respondents, the resulting analytic sample size was 373,580. The 2013 median American Association of Public Opinion Research response rate across the 50 states and DC was 45.9%.

Intake recommendations appropriate for adults who engage in <30 minutes of moderate physical activity daily are based on the *Dietary Guidelines for Americans* (1) and are expressed in cup equivalents, whereas BRFSS captures frequency of intake. To estimate the percentage of each state’s population meeting fruit and vegetable intake recommendations, previously developed prediction equations were applied to the frequency of intake data from BRFSS (6); these analyses are fully described elsewhere (6). In summary, 24-hour dietary recall data from the National Health and Nutrition Examination Survey (NHANES) for the period 2007–2010 were used to fit age- and sex-specific logistic regression models that estimate probabilities of meeting recommendations as functions of reported daily frequency of consumption, race/ethnicity, and income-to-poverty ratio, adjusting for day-to-day dietary variation. Reported daily frequencies of fruit and vegetable intake from BRFSS were calculated by dividing weekly frequencies by seven, monthly frequencies by 30, and yearly frequencies by 365. BRFSS respondents’ race/ethnicity (Hispanic, non-Hispanic black, and all others) and income-to-poverty ratio (<125%, 125%–349%, and ≥349%) were defined consistent with previous analyses (6). For income-to-poverty ratio, poverty was defined according to federal poverty guidelines.† Respondents’ reported daily frequencies of fruit juice and whole fruit intake, race/ethnicity, and income-to-poverty ratio were used as predictors in the models to estimate each respondent’s predicted probability of meeting the fruit intake recommendations. Reported daily intake frequencies of dried beans, dark green vegetables, orange vegetables, and other vegetables, along with demographic information, were used as predictors in the models to estimate probabilities of meeting vegetable intake recommendations. Predicted probabilities were weighted and averaged across all respondents and in each state to obtain the percentage of each state’s population meeting recommendations, using statistical software to account for the complex survey design. Balanced repeated replication technique, replicate weights, and Taylor linearization were used to compute standard errors and confidence intervals accounting for variation in the prediction models and BRFSS.

Median frequency of reported fruit intake across all respondents was once per day, ranging from 0.9 in Arkansas to 1.3 times per day in California (Table). Median frequency of reported vegetable intake was 1.7 times per day, ranging from 1.4 in Louisiana, Mississippi, and North Dakota to 1.9 times per day in California and Oregon. Based on prediction equations, 13.1% of respondents met fruit recommendations, and 8.9% met vegetable recommendations. The percentage of state populations meeting recommendations for fruits ranged from 7.5% in Tennessee to 17.7% in California, and for vegetables, from 5.5% in Mississippi to 13.0% in California.

## Discussion

In 2013, most adults consumed too few fruits and vegetables, with substantial variation by state. This analysis enhances current surveillance efforts by enabling the comparison of fruit and vegetable intake from the BRFSS survey module with federal recommendations. Ongoing collection of relevant state-level nutritional status and program data help identify public health nutrition problems in each state and support the design, evaluation, and management of nutrition intervention programs, in addition to catalyzing local interest in nutrition programs and policies (7).

Because fruit and vegetable consumption affects multiple health outcomes (1) and is currently low across all states, continued efforts are needed to increase demand and consumption. Improving fruit and vegetable consumption for adults might start with improving intake during childhood. During 2007–2010, 60% of children consumed fewer cup equivalents of fruit than recommended, and 93% consumed fewer vegetables than recommended (2). Better dietary practices earlier in life might lead to better practices later in life, and places where children learn and play can have an integral role in improving intake. For example, school districts, schools, and early care and education providers can help increase children’s fruit and vegetable consumption by meeting or exceeding current federal nutrition standards for meals and snacks, serving fruit and vegetables whenever food is offered, and training staff to make fruit and vegetables more appealing and accessible.§ Improving fruit and vegetable accessibility, placement, and promotion in grocery stores, restaurants, worksites, and other community settings might improve intake in adults (8,9). For example, work sites can make it easier for employees to make healthy food choices and create social norms that support healthy eating by creating policies to ensure that fruits and vegetables are provided at work-site gatherings, including meetings, conferences, and other events (8). CDC funds state, local, tribal, and territorial health departments to improve environments in worksites, schools, child care, and community settings to expand access to fruits and vegetables and other healthy food and beverage choices for persons of all ages.¶

The findings in this report are subject to at least five limitations. First, self-reports of intake are based on a limited set of questions and are prone to measurement error and recall bias (10). Self-reported intake might overestimate intake in some populations and underestimate intake in others (10). Second, these results might not be generalizable to the entire U.S. adult population (4). BRFSS excludes those living in nursing homes, long-term care facilities, military installations, and correctional institutions (4), but the overall effect this would have on the estimation of intake is unclear. Moreover, territories were excluded because prediction models were derived from NHANES, which excludes territories.** Third, estimates do not include non-100% fruit juice or fried potatoes because BRFSS respondents are instructed not to include them. Including these sources results in 4%–6% higher estimates for fruit and 30%–44% higher estimates for vegetables (6) but federal dietary guidelines recommend limiting foods and beverages with added sugars and solid fats (1). Fourth, relatively low response rates for BRFSS might have biased the sample. Finally, using prediction equations to estimate intake might have resulted in measurement error. However, previous analyses showed that applying prediction equations to 2011 BRFSS frequency data yielded estimates comparable to 2007–2010 national estimates that used more accurate 24-hour recalls (6).

**Summary**

What is already known about this topic?

Although national estimates indicate low fruit and vegetable intake, substantial variation by state has been observed. Fruit and vegetable intake information from the Behavioral Risk Factor Surveillance System (BRFSS) is the sole source of dietary information for most states, but the frequency of fruit and vegetable intake it captures cannot be directly compared to federal intake recommendations, which are expressed in cup equivalents.

What is added by this report?

CDC analyzed the percentage of each state’s population meeting fruit and vegetable intake recommendations from the most recent BRFSS survey for the 50 states and the District of Columbia, using a new scoring procedure. In 2013, 13.1% of respondents met fruit intake recommendations, ranging from 7.5% in Tennessee to 17.7% in California, and 8.9% met vegetable recommendations, ranging from 5.5% in Mississippi to 13.0% in California.

What are the implications for public health practice?

Substantial new efforts are needed to build consumer demand for fruits and vegetables through competitive pricing, placement, and promotion in child care, schools, grocery stores, communities, and worksites.

These results indicate that <18% of adults in each state consumed the recommended amount of fruit and <14% consumed the recommended amount of vegetables. Increased attention to food environments in multiple settings, including child care, schools, communities, and worksites, might help improve fruit and vegetable intake, and thus help prevent chronic disease.

## Figures and Tables

**TABLE:** State-specific frequency of fruit and vegetable intake among adults aged ≥18 years and percentage of respondents meeting federal fruit and vegetable intake recommendations — Behavioral Risk Factor Surveillance System, United States, 2013*

State†	No. in sample§	Median times consumed daily		% of respondents meeting recommendations¶			

				Fruit		Vegetables	
		
		Fruit	Vegetables	%	(95% CI)	%	(95% CI)
Overall	373,580	1.0	1.7	13.1	(12.0–14.2)	8.9	(5.8–12.0)
Alabama	4,613	1.0	1.6	9.5	(7.8–11.2)	7.1	(3.8–10.4)
Alaska	3,825	1.0	1.8	13.5	(11.4–15.6)	10.5	(6.2–14.8)
Arizona	3,269	1.0	1.6	12.5	(10.2–14.8)	9.8	(5.3–14.3)
Arkansas	3,914	0.9	1.5	9.4	(7.7–11.1)	7.5	(4.0–11.0)
California	9,011	1.3	1.9	17.7	(15.9–19.5)	13.0	(8.9–17.1)
Colorado	10,583	1.1	1.8	14.1	(12.5–15.7)	10.1	(6.4–13.8)
Connecticut	5,956	1.1	1.6	14.8	(12.9–16.7)	8.7	(5.1–12.3)
Delaware	4,015	1.0	1.5	12.8	(11.0–14.6)	7.5	(3.9–11.1)
District of Columbia	3,719	1.1	1.8	15.2	(12.9–17.5)	9.2	(4.7–13.7)
Florida	25,902	1.1	1.7	14.8	(13.2–16.4)	9.6	(6.3–12.9)
Georgia	5,993	1.0	1.6	11.7	(10.1–13.3)	8.1	(4.7–11.5)
Hawaii	6,549	1.0	1.7	12.4	(10.7–14.1)	10.2	(6.6–13.8)
Idaho	4,518	1.0	1.7	12.3	(10.3–14.3)	8.9	(4.9–12.9)
Illinois	5,016	1.1	1.6	14.6	(12.6–16.6)	8.7	(4.9–12.5)
Indiana	7,821	1.0	1.5	11.4	(9.9–12.9)	7.3	(4.0–10.6)
Iowa	6,500	1.0	1.5	11.3	(9.8–12.8)	6.6	(3.2–10.0)
Kansas	18,535	1.0	1.6	10.4	(9.2–11.6)	8.3	(5.0–11.6)
Kentucky	6,959	1.0	1.6	9.5	(8.0–11.0)	7.1	(3.7–10.5)
Louisiana	3,839	1.0	1.4	9.8	(8.0–11.6)	6.9	(3.3–10.5)
Maine	6,697	1.1	1.8	14.5	(12.7–16.3)	9.6	(6.4–12.8)
Maryland	9,817	1.1	1.7	13.2	(11.6–14.8)	8.4	(4.9–11.9)
Massachusetts	11,295	1.1	1.7	14.2	(12.6–15.8)	9.4	(5.9–12.9)
Michigan	10,263	1.0	1.6	12.7	(11.2–14.2)	7.7	(4.4–11.0)
Minnesota	11,491	1.0	1.6	12.5	(10.8–14.2)	7.9	(4.4–11.4)
Mississippi	5,567	1.0	1.4	9.9	(8.3–11.5)	5.5	(2.3–8.7)
Missouri	5,435	1.0	1.6	10.5	(8.9–12.1)	7.8	(4.2–11.4)
Montana	8,023	1.0	1.7	12.2	(10.6–13.8)	9.2	(5.6–12.8)
Nebraska	14,004	1.0	1.6	12.3	(10.7–13.9)	8.3	(4.8–11.8)
Nevada	3,957	1.0	1.7	14.0	(11.7–16.3)	10.3	(6.0–14.6)
New Hampshire	5,040	1.1	1.7	14.8	(12.8–16.8)	9.9	(6.3–13.5)
New Jersey	9,812	1.1	1.7	13.4	(11.9–14.9)	8.3	(5.0–11.6)
New Mexico	7,326	1.0	1.7	12.1	(10.5–13.7)	10.0	(6.0–14.0)
New York	6,796	1.1	1.7	15.5	(13.7–17.3)	8.8	(5.1–12.5)
North Carolina	6,396	1.0	1.7	10.3	(8.8–11.8)	7.2	(3.9–10.5)
North Dakota	6,206	1.0	1.4	11.4	(9.7–13.1)	6.4	(2.4–10.4)
Ohio	9,285	1.0	1.5	11.3	(9.8–12.8)	7.1	(3.9–10.3)
Oklahoma	6,594	1.0	1.5	8.2	(6.9–9.5)	5.8	(2.4–9.2)
Oregon	4,556	1.1	1.9	14.5	(12.5–16.5)	11.0	(7.1–14.9)
Pennsylvania	8,756	1.0	1.6	12.7	(11.1–14.3)	7.5	(4.3–10.7)
Rhode Island	4,878	1.1	1.7	13.9	(12.0–15.8)	8.7	(5.0–12.4)
South Carolina	8,224	1.0	1.6	11.6	(10.1–13.1)	6.8	(3.5–10.1)
South Dakota	5,398	1.0	1.6	10.3	(8.5–12.1)	6.8	(3.1–10.5)
Tennessee	3,522	1.0	1.6	7.5	(6.0–9.0)	6.2	(2.7–9.7)
Texas	7,925	1.0	1.7	11.0	(9.5–12.5)	8.4	(4.2–12.6)
Utah	10,167	1.1	1.7	13.8	(12.1–15.5)	9.4	(5.2–13.6)
Vermont	5,136	1.1	1.8	14.5	(12.6–16.4)	10.8	(7.3–14.3)
Virginia	6,571	1.1	1.7	13.4	(11.7–15.1)	8.8	(5.2–12.4)
Washington	9,084	1.0	1.8	12.3	(10.8–13.8)	9.9	(6.3–13.5)
West Virginia	4,629	1.0	1.6	7.7	(6.4–9.0)	6.6	(3.6–9.6)
Wisconsin	5,212	1.1	1.5	12.7	(10.8–14.6)	7.5	(3.6–11.4)
Wyoming	4,981	1.0	1.7	11.9	(10.1–13.7)	9.4	(5.5–13.3)

**Abbreviation:** CI = confidence interval.

* Estimates are weighted to account for complex sampling using statistical software except where noted. Fruit consists of 100% fruit juice and whole fruit. Vegetables include dried beans, dark green vegetables, orange vegetables, and other vegetables.

† Includes the District of Columbia.

§ Number of respondents (unweighted) with complete data for fruit and vegetable intake and demographic information.

¶ Recommendations are age- and sex-specific and are appropriate for adults who engage in <30 minutes of moderate physical activity daily, beyond normal daily activities. Percentages are derived from age- and sex-specific models that account for the usual intake of foods, race/ethnicity, and income-to-poverty ratio. Additional information available at http://www.choosemyplate.gov/printpages/MyPlateFoodGroups/Fruits/food-groups.fruits-amount.pdf and http://www.choosemyplate.gov/printpages/MyPlateFoodGroups/Vegetables/food-groups.vegetables-amount.pdf.

## Footnotes

* Those who are more physically active might be able to consume more while staying within calorie needs. Additional information available at http://www.choosemyplate.gov/printpages/MyPlateFoodGroups/Fruits/food-groups.fruits-amount.pdf and http://www.choosemyplate.gov/printpages/MyPlateFoodGroups/Vegetables/food-groups.vegetables-amount.pdf.

† Additional information available at http://aspe.hhs.gov/poverty/13poverty.cfm.

§ Additional information available at http://www.cdc.gov/vitalsigns/fruit-vegetables.

¶ Additional information available at http://www.cdc.gov/chronicdisease/about/state-public-health-actions.htm, http://www.cdc.gov/chronicdisease/about/foa/2014foa/index.htm, http://www.cdc.gov/nccdphp/dch/programs/index.htm, and http://www.cdc.gov/nccdphp/dnpao/state-local-programs/index.html.

** Guam and Puerto Rico were the only two territories to collect fruit and vegetable intake data in BRFSS in 2013. If NHANES is representative of territorial populations, 14% and 11% of the population met fruit intake recommendations in Guam and Puerto Rico, respectively, and 11% and 4% met vegetable intake recommendations.
